# Identification of Genes Preferentially Expressed by Highly Virulent Piscine *Streptococcus agalactiae* upon Interaction with Macrophages

**DOI:** 10.1371/journal.pone.0087980

**Published:** 2014-02-03

**Authors:** Chang-Ming Guo, Rong-Rong Chen, Dildar Hussain Kalhoro, Zhao-Fei Wang, Guang-Jin Liu, Cheng-Ping Lu, Yong-Jie Liu

**Affiliations:** College of Veterinary Medicine, Nanjing Agricultural University, Nanjing, China; Columbia University, United States of America

## Abstract

*Streptococcus agalactiae*, long recognized as a mammalian pathogen, is an emerging concern with regard to fish. In this study, we used a mouse model and *in vitro* cell infection to evaluate the pathogenetic characteristics of *S. agalactiae* GD201008-001, isolated from tilapia in China. This bacterium was found to be highly virulent and capable of inducing brain damage by migrating into the brain by crossing the blood–brain barrier (BBB). The phagocytosis assays indicated that this bacterium could be internalized by murine macrophages and survive intracellularly for more than 24 h, inducing injury to macrophages. Further, selective capture of transcribed sequences (SCOTS) was used to investigate microbial gene expression associated with intracellular survival. This positive cDNA selection technique identified 60 distinct genes that could be characterized into 6 functional categories. More than 50% of the differentially expressed genes were involved in metabolic adaptation. Some genes have previously been described as associated with virulence in other bacteria, and four showed no significant similarities to any other previously described genes. This study constitutes the first step in further gene expression analyses that will lead to a better understanding of the molecular mechanisms used by *S. agalactiae* to survive in macrophages and to cross the BBB.

## Introduction

*Streptococcus agalactiae*, commonly known as group B streptococcus (GBS), has a broad host range and is pathogenic to mammals, reptiles, amphibians, and fish [1]. It has been reported to cause neonatal pneumonia and meningitis in humans, mastitis in cows and meningoencephalitis in fish [2], [3], [4]. Recently, numerous outbreaks of *S. agalactiae* infections have been described in multiple fish farms, especially tilapia farms [5], [6], [7]. Since 2009, an outbreak of severe infectious GBS disease has occurred in tilapia culture farms in the south of China, causing large economic losses due to high mortality in the infected fish [8].

The pathogenesis of *S. agalactiae* infection in tilapias is not yet fully described or understood. It is known that once GBS injures or penetrates cellular barriers to reach the bloodstream or deeper tissues, an immunologic response is triggered to clear the organism. Central to this response are host phagocytic cells, including neutrophils and macrophages [9]. The ability of GBS to remain in the host as a commensal organism and to establish infection in susceptible individuals suggests that the organism may be able to subvert the host immune system [10]. It has been reported that when GBS is engulfed by professional phagocytic cells, such as macrophages and neutrophils, the organism can remain viable for a long period of time [11], [12], [13], although the mechanism of survival is unknown. The intracellular localization of GBS in macrophages may protect the organism from more active antimicrobial molecules in the blood and thus may be important in establishing bacteremia and subsequent meningitis. In line with this, in the closely related organism and extracellular pathogen *Streptococcus pyogenes*, it has been suggested that an important mechanism to establish infection is the survival of the organism in phagocytic cells [14]. Macrophages have been suggested to serve as vectors in the blood-borne spread of *Listeria monocytogenes* to cerebral endothelial cells, followed by further spread into the brain parenchyma [15], [16], [17], [18]. An early “Trojan Horse” theory suggested that *Streptococcus suis* is taken up by macrophages, allowing the bacteria to survive and travel intracellularly in the circulation [19].

One way to understand host-pathogen interactions is to dissect their molecular mechanisms by studying gene expression [20]. Selective capture of transcribed sequences (SCOTS) is a PCR-based RNA analytical method that has been used with success in many bacteria [20], [21], [22], [23]. In this study, we investigated the virulence of *S. agalactiae* isolated from fish using animal and cell culture models and used the SCOTS approach to identify genes preferentially expressed by *S. agalactiae* upon interactions with murine macrophages RAW264.7, a process that might be essential for the establishment of infection by this pathogen. To our best knowledge, this is the first report to broadly define transcripts expressed by *S. agalactiae* in macrophages.

## Results

### Experimental infection of mice

To determine the virulence of *S. agalactiae* GD201008-001, we performed the bacterial infection in BALB/c mice. Surprisingly, all mice injected intraperitoneally with 10^1^–10^4^ CFU in 100 µl of PBS died within 36 h post infection, while no mice died in the PBS control group (Table 1). It suggests that the bacterial strain GD201008-001 is highly virulent to BALB/c mice by intraperitoneal administration, with LD_50_ values of less than 10 CFU.

**Table 1:** Determination of LD50 in BALB/c mice challenged with *S. agalactiae* GD201008-001.

Dose of challenge CFU/(0.1 ml)	Number of death/total		
	12 h.p.i.	24 h.p.i.	36 h.p.i.
1×10^4^	0/10	10/10	10/10
1×10^3^	0/10	8/10	10/10
1×10^2^	0/10	6/10	10/10
1×10^1^	0/10	5/10	10/10
PBS	0/10	0/10	0/10
LD50	<1×10^1^CFU		

**Figure 1:**
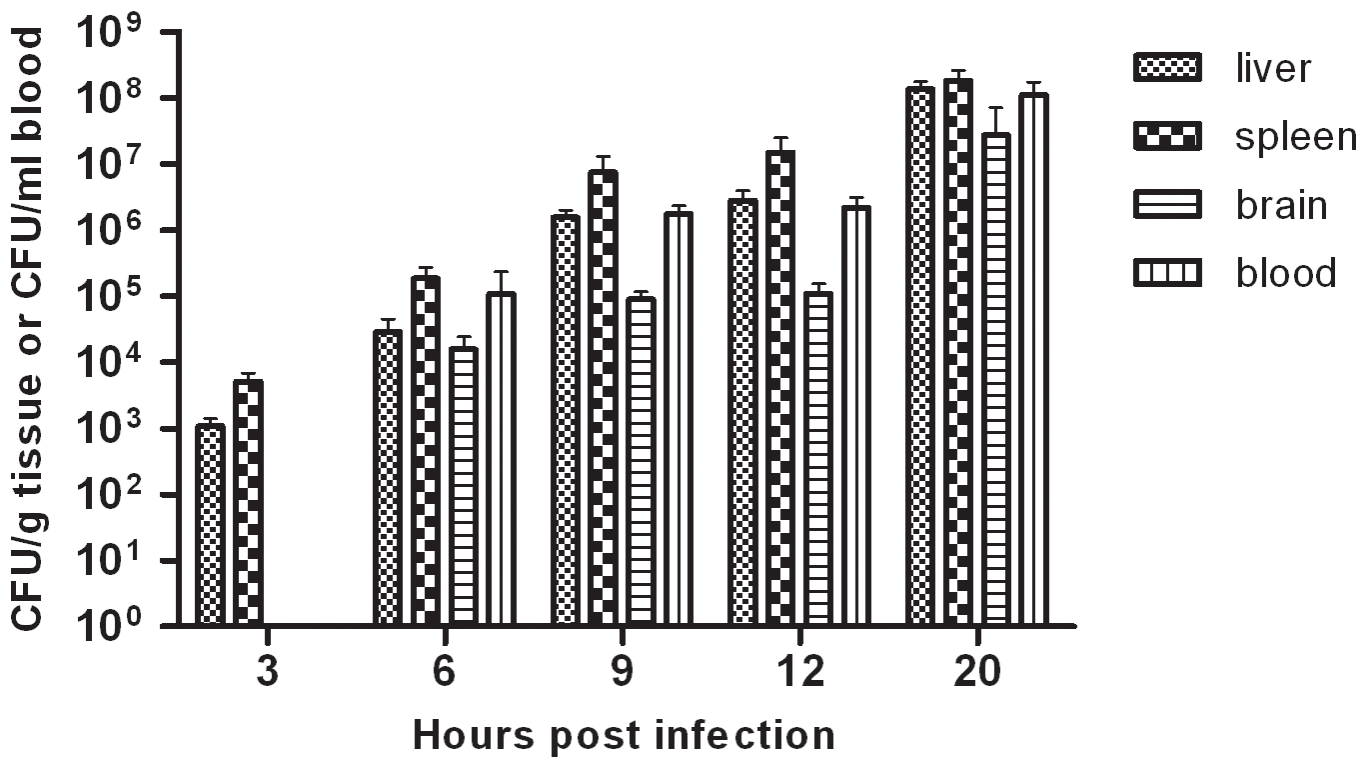
Recovery of bacteria from liver, spleen, brain and blood of mice infected with GD201008-001. Recovered bacteria are expressed as CFU per gram of liver, spleen or brain and CFU per milliliter of blood.

Twenty-five mice were inoculated intraperitoneally with 2×10^2^ CFU of the bacterial strain in 100 µl of PBS. Bacterial isolation was carried out to determine bacterial loads in the main tissues from infected mice (Fig. 1).

At 3 hours post-infection (h.p.i.), bacterial loads exceeded 10^3^ CFU/g in liver and spleen whilst this bacterium could not be isolated from brains or blood at this time. Three hours after, at 6 h.p.i, bacterial loads exceeded 10^4^ CFU/ml in blood and 10^4^ CFU/g in liver, spleen and brain. Bacterial number continued to increase with time over the course of the infection. At 20 h.p.i., bacterial loads were more than 10^8^ CFU/ml in blood, 10^8^ CFU/g in liver and spleen, and more than 10^7^ CFU/g in the brain.

### Histopathology and immunohistochemistry for brain tissues

To determine the pathological damage to infected mouse brains, we chose brains from infected mice at 20 h.p.i. to perform histopathological and immunohistochemical analyses.

**Figure 2:**
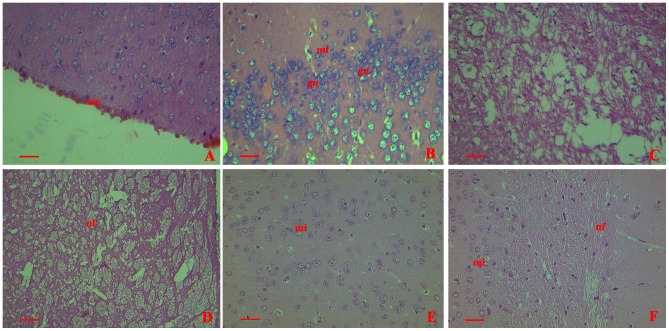
Histopathological changes shown by H&E staining in collected brains from mice infected with GD201008-001 at 20 h.p.i. me = meninges;mi = microglia;gn =  glial nodules;hc = Hippocampus;nf = nerve fibers. (A) Prominent meningeal hemorrhage and erythrocyte aggregation in the meninges (400×). (B) Microglial cells showing an increase in the number and volume. Glial nodules were distributed (400×). (C) Lytic and necrotic hippocampus (400×). (D) Nerve fibers showing severe damage (400×). (E) and (F) No histopathological changes in sham infection control (injected with PBS) (400×). Scale bar  =  20 µm.

Histopathologically, all the brains from infected mice showed significant lesions. Meningeal hemorrhage and erythrocyte aggregation were prominent (Fig. 2A). Microglial cell numbers increased and cell volumes enlarged significantly. Also, glial nodules were found (Fig. 2B). The hippocampus was lytic and necrotic (Fig. 2C). And signs of damage were apparent in the nerve fibers (Fig. 2D). No alterations were found in the brains from the control group (Fig. 2E and Fig. 2F).

**Figure 3:**
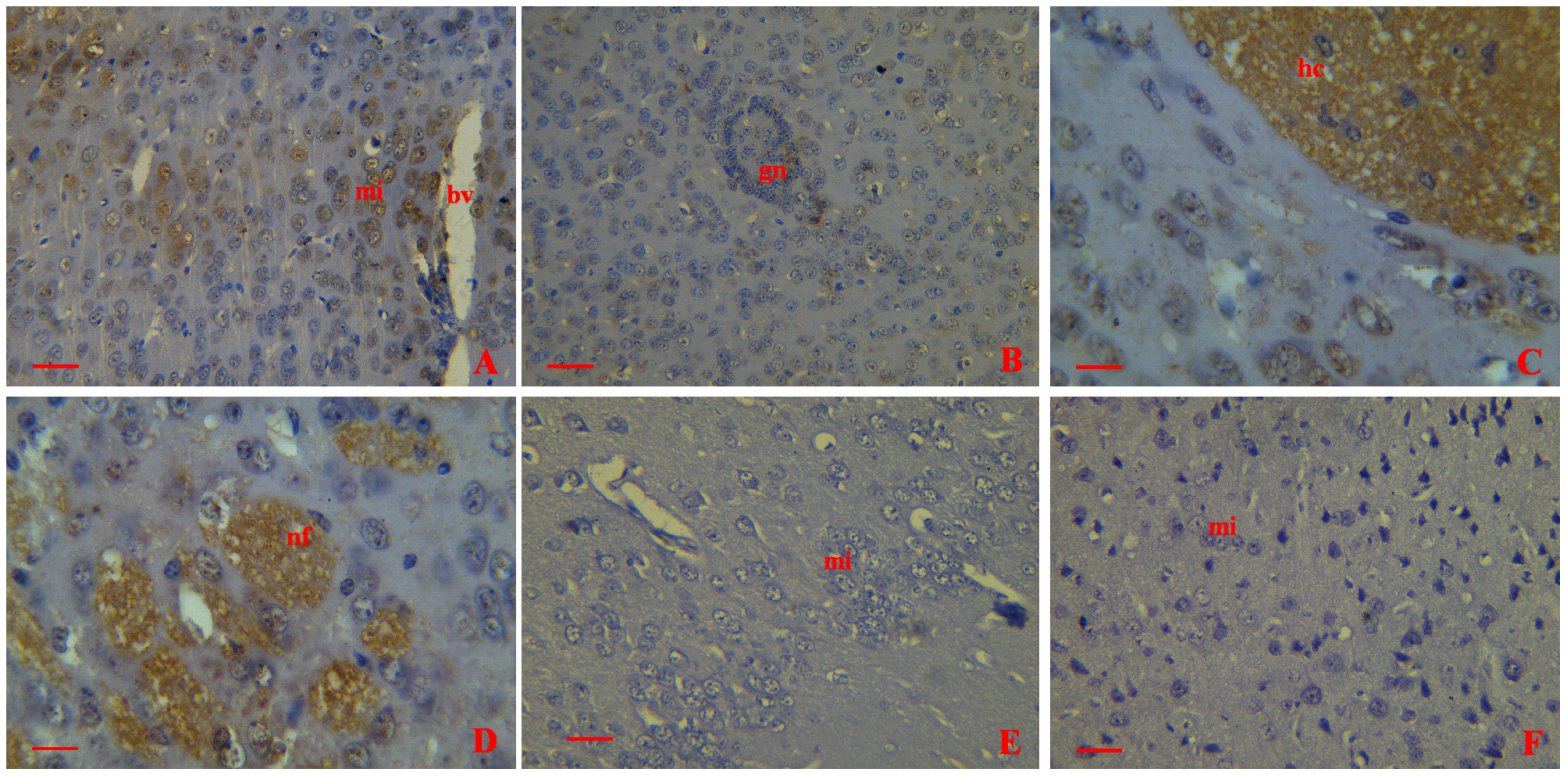
Immunohistochemical detection of bacterial antigen in collected brains from mice infected with GD201008-001at 20 h.p.i.. bv = blood vessel;mi = microglia;gn =  glial nodules;hc = Hippocampus;nf = nerve fibers (A) Positive stain in the microglia (400×). (B) Bacterial antigens were detected in the glial nodules (400×). (C) Intense antigen staining in the hippocampus. (D) A large amount of bacterial antigens were detected in the nerve fibers (400×). (E) Negative control (injected with GBS, stained with unimmunized rabbit serum) showed clean background (400×). (F) Sham mice (injected with PBS, stained with immunized rabbit serum) showed no antigen staining (400×). Scale bar  =  20 µm.

Using immunohistochemistry, bacterial antigens were demonstrated in the brains of all infected mice. Antigen could be detected in the microglia. The closer to the blood vessel the microglias were, the more antigens were found inside the cells (Fig. 3A). Antigen staining was also found in the glial nodules (Fig. 3B). A large amount of bacterial antigens were detected in the hippocampus (Fig. 3C) and nerve fibers (Fig. 3D). No evidences of positive staining were found in the negative control group (injected with GBS, stained with unimmunized rabbit serum) (Fig. 3E) and sham infection control group (injected with PBS, stained with immunized rabbit serum) (Fig. 3F).

### *S. agalactiae* interactions with murine macrophages

**Figure 4:**
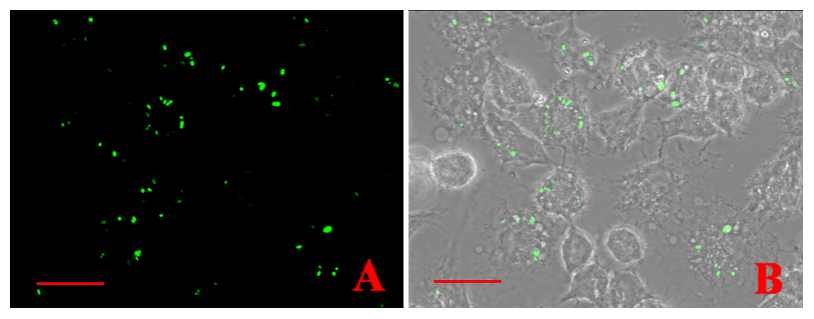
Fluorescence microscopy-based phagocytosis of GFP- expressing *S. agalactiae*. GFP expressing bacteria were internalized by RAW264.7(1000×). Scale bar  =  20 µm.

Temporal observation of the behavior of *S. agalactiae* GD201008-001 after phagocytosis by RAW264.7 was made using GFP as an intrinsic label to track the bacterial cells following ingestion by macrophages. These observations revealed that the GFP-expressing *S. agalactiae* could be visualized within the macrophages (Fig. 4).

**Figure 5:**
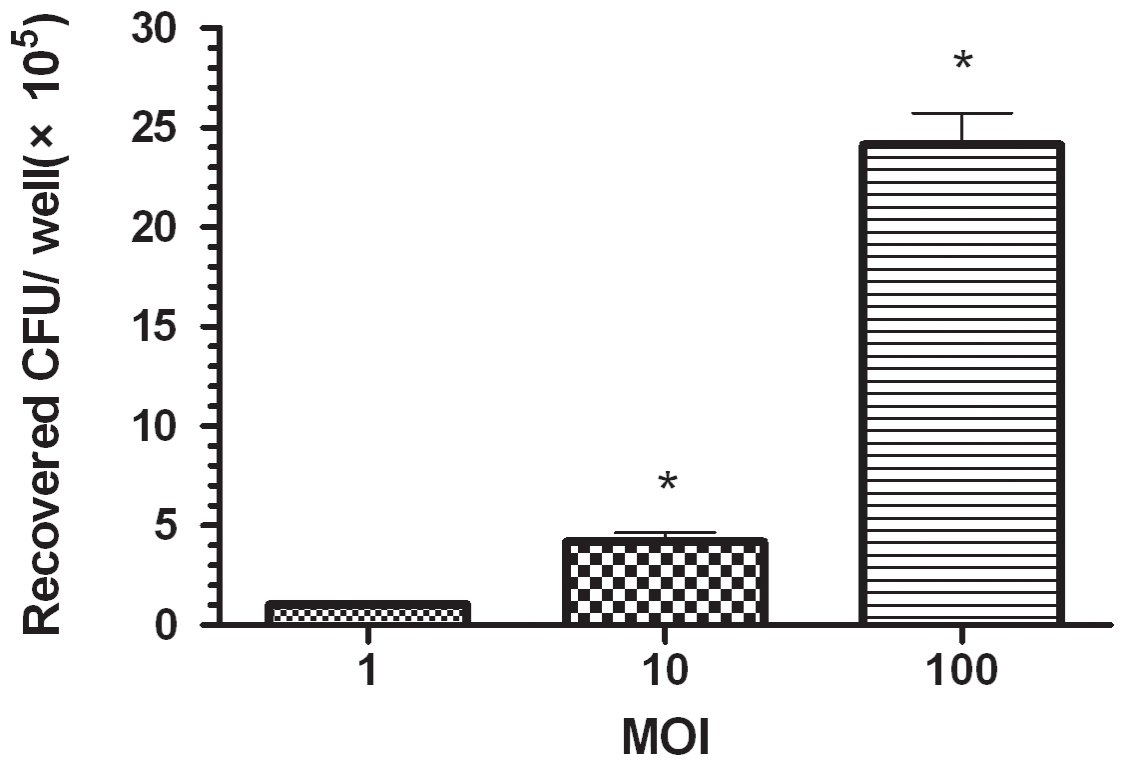
Phagocytosis of GD201008-001 by RAW264.7 macrophages. At 1(means ± S.D. obtained from three independent experiments, n = 4). An asterisk indicates a significant difference versus the phagocytosis values from the group treated at an MOI of 1 (P<0.05).

To determine the ability of macrophages to internalize *S. agalactiae*, RAW264.7 macrophages were infected at MOI = 1, 10 or 100 with *S. agalactiae* for 1 h. After antibiotic treatment to kill the extracellular bacteria, intracellular bacteria were plated onto Todd-Hewitt agar to be cultured overnight. As shown in Fig. 5, we were able to show that entry of *S. agalactiae* into RAW264.7 macrophages occurs in a dose-dependent manner. The MOI = 10 and MOI = 100 groups were significantly (p<0.05) more internalized by macrophages than bacteria at MOI = 1.

**Figure 6:**
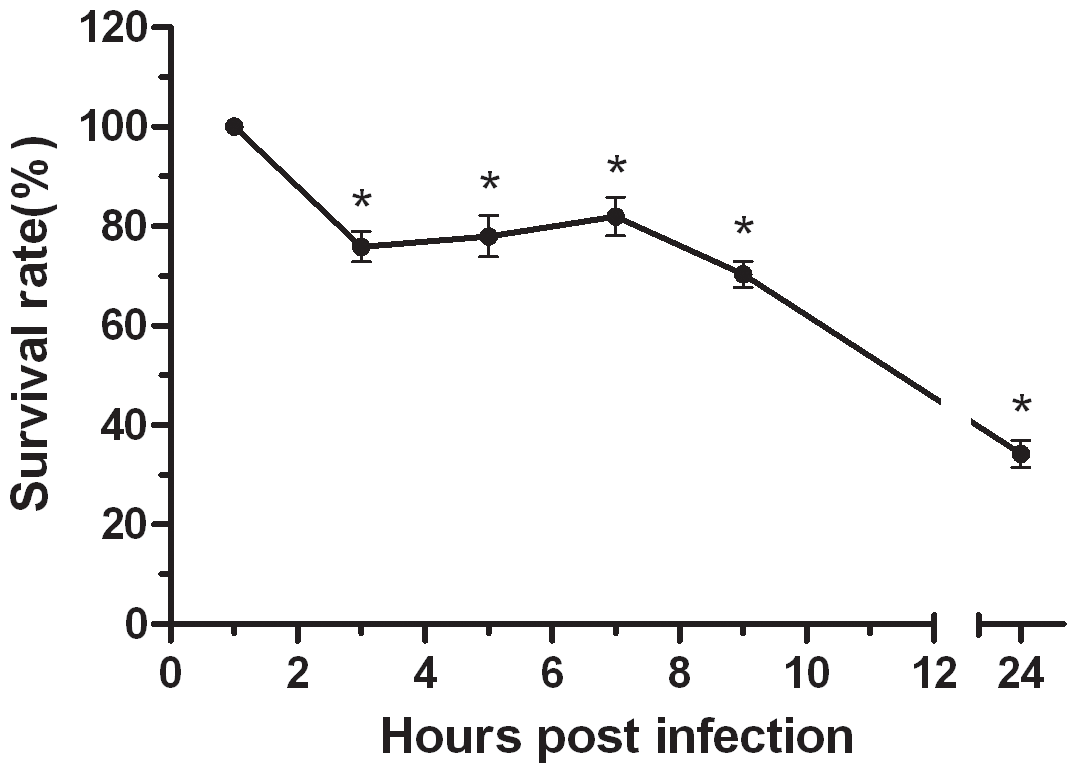
Intracellular survival of *S. agalactiae* within RAW264.7 macrophages. RAW264.7 cells were infected with *S. agalactiae* (MOI = 1) and phagocytosis was left to proceed for 1 h. Antibiotics were then added for a period of 1 h. This initial antibiotic-treatment was extended for different times up to 24 h and cells were lysed to quantify the intracellular bacteria by viable plate counting. The results are expressed as the intracellular survival rates of the bacteria (means± SD obtained from three independent experiments, n = 4). An asterisk indicates a significant difference versus the intracellular bacteria survival rate of the 1 h.p.i group (P<0.05).

To analyze the intracellular fate of the bacteria once internalized, we modified the phagocytosis assay in order to quantify intracellular bacterial survival over time. After exactly 60 min of incubation of *S. agalactiae* with macrophages to allow internalization, antibiotics were added and the treatment was lengthened for different times up to 24 h. As shown in Fig. 6, after 60 min of phagocytosis, the numbers of viable intracellular bacteria showed an obvious decrease within phagocytic cells over the first 3 h.p.i., then increased slightly up to 7 h.p.i, but then descended again. Finally the numbers of viable intracellular bacteria rate decreased to about 34% of their maximum at 24 h.p.i.

**Figure 7:**
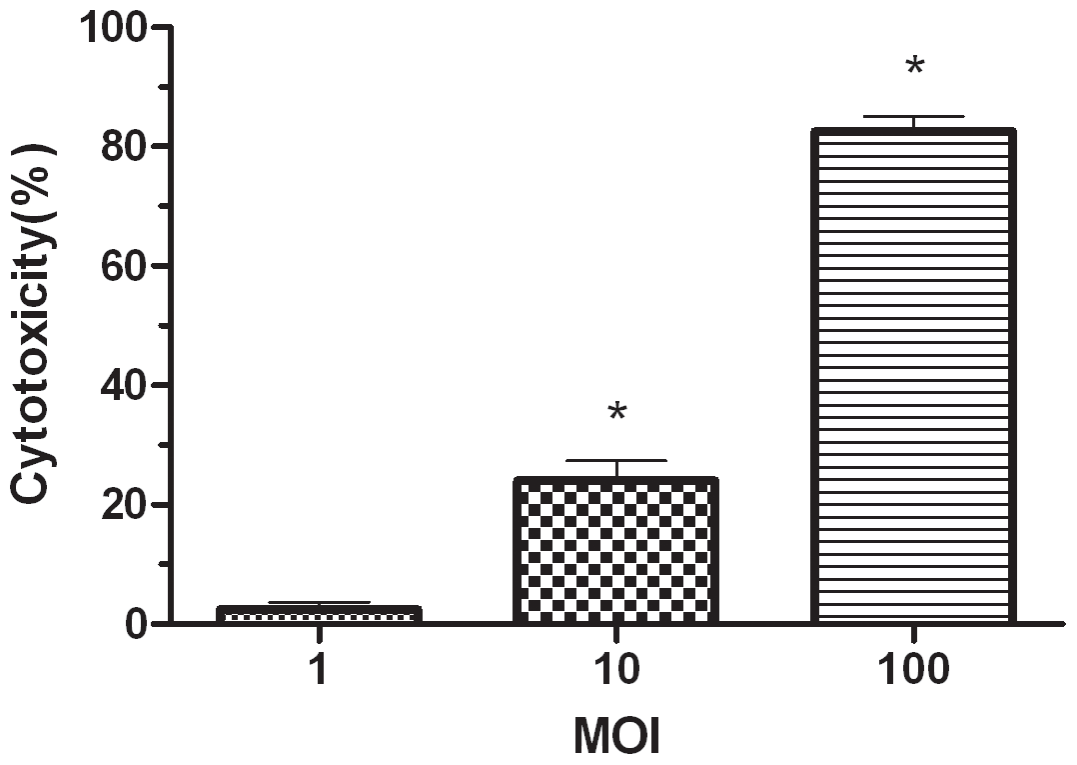
Cytotoxic effect of *S. agalactiae* on RAW264.7 macrophages. The percentage of macrophage cytotoxicity was determined by measuring LDH release in the presence of different concentrations (MOI of 1, 10 or 100) of *S. agalactiae* GD201008-001 after a 4 h incubation at 37°C. An asterisk indicates that the bacterial cytotoxicity is significantly different (p<0.05) from the cytotoxicity value at an MOI of 1. (n = 4).

In order to determine if *S. agalactiae* could be cytotoxic to RAW264.7, lactose dehydrogenase (LDH) release was measured. As shown in Fig. 7, GD201008-001 was found to be cytotoxic to macrophages in a concentration-dependent manner. At an MOI of 1, the cells showed almost no toxicity, even at 4 h.p.i. However, in contrast to the MOI = 1 group, the cytotoxicity levels at MOIs of 10 and 100 were significantly higher (P<0.05). In particular, the level of cytotoxicity was up to 82% at an MOI of 100.

### Selective capture of *S. agalactiae* transcripts from infected macrophages

**Figure 8:**
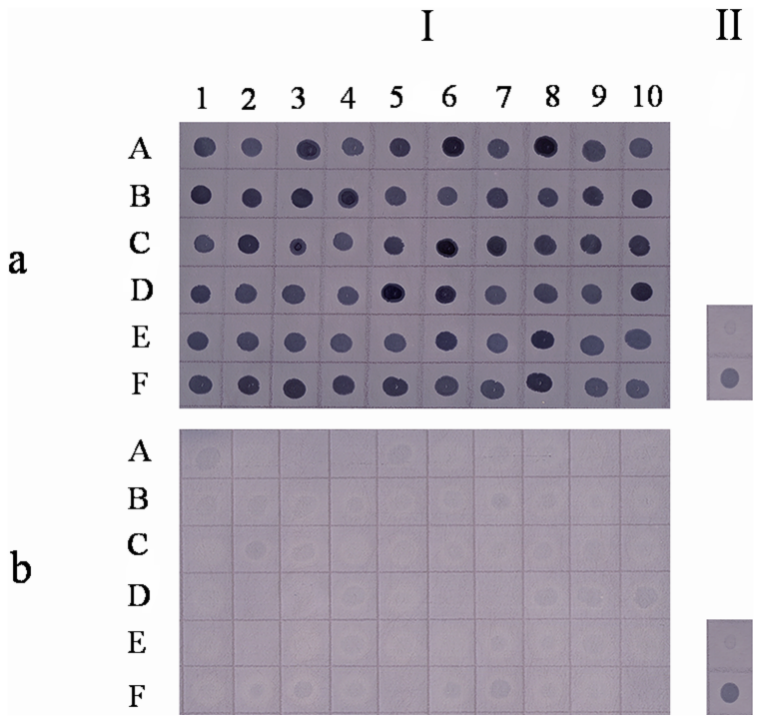
Southern dot blot analysis of selected SCOTS clones. Southern dot blot showing (I) SCOTS clones (each dot corresponds to one clone in Table 2) and (II) 0.1 µg (top) or 1 µg (bottom) of genomic *S. agalactiae* DNA as positive controls, hybridized to a digoxigenin (DIG)-labeled probe generated from RAW264.7-derived (a) or culture-derived cDNA (b) after three rounds of normalization.

To identify *S. agalactiae* genes preferentially expressed under interaction with macrophages using the SCOTS approach, a total of sixty clones that hybridized to a cDNA probe from *S. agalactiae* surviving in macrophages but did not hybridize to a cDNA probe prepared from bacteria grown in culture medium, were chosen for further characterization analysis (Fig. 8). Among the 60 clones, four showed no homology to genes with known function. The remaining 56 genes could be characterized into 6 functional categories (Table 2): (1) Nine genes were involved in cell envelope biogenesis/outer membrane synthesis, responsible for the construction of peptidoglycans, cell surface proteins, or components of bacterial secretory systems. Among these, *dltA* and clone 20 (D-alanyl-D-alanine carboxypeptidase) were associated with cell wall biogenesis and four genes belonged to cell membrane proteins; (2) Thirty-two genes were involved in metabolism/stress response, including enzymes involved in the biosynthesis and metabolism of sugar, fatty acids, proteins, and nucleic acids. The up-regulated genes were an adaptation to oxidative stress inside the macrophages and an environment lacking nutritional resources; (3) Four transporters genes were identified, including the components of ABC-type transport systems and chloride channels; (4) Five genes were involved in cell division/replication, including genes associated with the replication and genetic regulation of the bacteria. *DivIB* and *rpoC* were involved in cell replication. *UvrA* was involved in cell damage repair; (5) Three genes were involved in protein sorting, including molecular chaperones and signal peptidase; (6) Four genes possessed regulatory functions, including transcriptional regulation. Examples included *hrcA,* encoding a heat-inducible transcription repressor, and *covR,* encoding the response regulator of the two-component CovR/S regulatory system.

**Table 2:** Genes identified by SCOTS that are differentially expressed by *S. agalactiae* upon interaction with RAW264.7.

Function and clone	Gene and/or possible function	GenBank Identification	Reference(s)
**Cell envelope biogenesis, outer membrane**			
Clone A1	*cfa*,	YP_330368.1	
	cyclopropane-fatty-acyl-phospholipid synthase (*S.agalactiae* A909)		
Clone A2	*dltA*,	ZP_08649940.1	[29], [30], [31], [32], [33], [34]
	D-alanine-D-alanyl carrier protein ligase subunit 1 (*S.agalactiae* ATCC 13813)		
Clone A3	D-alanyl-D-alanine carboxypeptidase (*S. agalactiae* 2603V/R)	NP_687183.1	
Clone A4	cell wall surface anchor family protein (*S.agalactiae* A909)	YP_329518.1	
Clone A5	*secA*,	ZP_08650039.1	[35], [36]
	preprotein translocase subunit SecA (*S.agalactiae* ATCC 13813)		
Clone A6	polysaccharide biosynthesis protein (*S.agalactiae* A909)	YP_330055.1	
Clone A7	*cstA*,	NP_735499.1	
	hypothetical protein gbs1050 (*S. agalactiae* NEM316)		
Clone A8	hypothetical protein FSLSAGS3026_12085 (*S. agalactiae* FSL S3-026)	EGS27102.1	
Clone A9	Can B domain-containing protein (*S.agalactiae* FSL S3-026)	EGS27960.1	
**Metabolism/stress response**			
Clone A10	hypothetical protein SAG0894 (*S. agalactiae* 2603V/R)	NP_687908.1	
Clone B1	*hylB,*	YP_329897.1	[9]
	hyaluronate lyase (*S. agalactiae* A909)		
Clone B2	*clpE*,	NP_734999.1	[40], [41]
	hypothetical protein gbs0535 (*S. agalactiae* NEM316)		
Clone B3	peptidase, M20/M25/M40 family (*S. agalactiae* H36B)	ZP_00784166.1	
Clone B4	metallopeptidase, zinc binding (*S. agalactiae* COH1)	ZP_00784711.1	
Clone B5	endopeptidase O, putative (*S. agalactiae* CJB111)	ZP_00788097.1	
Clone B6	hypothetical protein SAG0872 (*S. agalactiae* 2603V/R)	NP_687886.1	
Clone B7	*pulA*,	ZP_00786133.1	[46], [47], [48], [49], [50]
	pullulanase, putative (*S. agalactiae* COH1)		
Clone B8	mannosyl-glycoprotein endo-beta-N-acetylglucosamidase family protein (*S. agalactiae* FSL S3-026)	EGS28355.1	
Clone B9	*ciaH*,	NP_735468.1	[37], [38], [39]
	sensor histidine kinase CiaH (*S. agalactiae* NEM316)		
Clone B10	*serB*,	NP_687649.1	
	phosphoserine phosphatase SerB (*S. agalactiae* 2603V/R)		
Clone C1	acetyltransferase (*S. agalactiae* 2603V/R)	NP_688337.1	
Clone C2	pyridine nucleotide-disulfide oxidoreductase family protein (*S. agalactiae* 2603V/R)	NP_688351.1	
Clone C3	*htpX*,	NP_688618.1	
	heat shock protein HtpX (*S. agalactiae* 2603V/R)		
Clone C4	CsbD family protein (*S. agalactiae* A909)	YP_329332.1	
Clone C5	*fhs*,	YP_329765.1	
	formate—tetrahydrofolate ligase (*S. agalactiae* A909)		
Clone C6	*folP*,	YP_329821.1	
	dihydropteroate synthase (*S. agalactiae* A909)		
Clone C7	*aroA*,	ZP_08650982.1	
	3-phosphoshikimate-1-carboxyvinyltransferase (*S. agalactiae* ATCC 13813)		
Clone C8	*nox*,	ZP_00783526.1	[42]
	NADH oxidase (*S. agalactiae* H36B)		
Clone C9	morphine 6-dehydrogenase (*S. agalactiae* ATCC 13813)	ZP_08650234.1	
Clone C10	*carB*,	ZP_08650601.1	[43]
	carbamoyl-phosphate synthase, large subunit (*S. agalactiae* ATCC 13813)		
Clone D1	*msrB*,	ZP_00785672.1	[44], [45]
	methionine sulfoxide reductases B (*S. agalactiae* COH1)		
Clone D2	*nrdI*,	YP_330497.1	
	flavoprotein NrdI (*S. agalactiae* A909)		
Clone D3	*pyrH*,	NP_688507.1	
	uridylate kinase (*S. agalactiae* 2603V/R)		
Clone D4	*sdhA*,	YP_330694.1	
	L-serine dehydratase, iron-sulfur-dependent subunit alpha (*S. agalactiae* A909)		
Clone D5	*aroD*, 3-dehydroquinate dehydratase (*S. agalactiae* 2603V/R)	NP_688377.1	
Clone D6	hypothetical protein SAK_0322 (*S. agalactiae* A909)	YP_328974.1	
Clone D7	*fabK*,	NP_687380.1	
	enoyl-ACP reductase (*S. agalactiae* 2603V/R)		
Clone D8	*cylI*,	EGS28236.1	
	cylI protein (*S. agalactiae* FSL S3-026)		
Clone D9	*gap*,	NP_688758.1	[51], [52]
	glyceraldehyde-3-phosphate dehydrogenase (*S. agalactiae* 2603V/R)		
Clone D10	glycosyl hydrolase (*S. agalactiae* A909)	YP_329453.1	
Clone E1	*ccpA*,	YP_329455.1	[53]
	catabolite control protein A (*S. agalactiae* A909)		
**Transport**			
Clone E2	voltage-gated chloride channel family protein (*S. agalactiae* 2603V/R)	NP_687570.1	
Clone E3	ABC transporter ATP-binding protein (*S. agalactiae* 2603V/R)	NP_689021.1	
Clone E4	oligopeptide ABC transporter oligopeptide-binding protein (*S. agalactiae* 2603V/R)	NP_687222.1	
Clone E5	thiW protein (*S. agalactiae* ATCC 13813)	ZP_08650771.1	
**Cell division/replication**			
Clone E6	*divIB*,	NP_687507.1	
	cell division protein DivIB (*S. agalactiae* 2603V/R)		
Clone E7	*uvrA*,	ZP_00783998.1	
	excinuclease ABC, A subunit (*S. agalactiae* H36B)		
Clone E8	MutS2 family protein (*S. agalactiae* FSL S3-026)	EGS26693.1	
Clone E9	IS861, transposase OrfB (*S. agalactiae* 2603V/R)	NP_688077.1	
Clone E10	*rpoC*,	EGS28475.1	
	DNA-directed RNA polymerase subunit beta (*S. agalactiae* FSL S3-026)		
**Protein sorting**			
Clone F1	*dnaJ*,	ZP_00783391.1	
	dnaJ protein (*S. agalactiae* H36B)		
Clone F2	*lepB*,	ZP_00788470.1	
	Signal peptidase I (*S. agalactiae* CJB111)		
**Regulatory**			
Clone F3	*hrcA*,	YP_328819.1	
	heat-inducible transcription repressor (*S. agalactiae* A909)		
Clone F4	MerR family transcriptional regulator (*S. agalactiae* A909)	YP_330526.1	[54]
Clone F5	LysR family transcriptional regulator (*S. agalactiae* NEM316)	NP_735874.1	[55]
Clone F6	*covR*,	YP_004478477.1	[10]
	response regulator protein (*S. parauberis* KCTC 11537)		
**Unknown function**			
Clone F7	unknown (*S. agalactiae* H36B)	ZP_00783887.1	
Clone F8	hypothetical protein SAG1491 (*S. agalactiae* 2603V/R)	NP_688485.1	
Clone F9	*yobC*,	AAL96904.1	
	hypothetical protein spyM18_0084 (*S.pyogenes* MGAS8232)		
Clone F10	hypothetical protein FSLSAGS3026_02113 (*S.agalactiae* FSL S3-026)	EGS28676.1	

### Validation of SCOTS results by q-PCR

**Figure 9:**
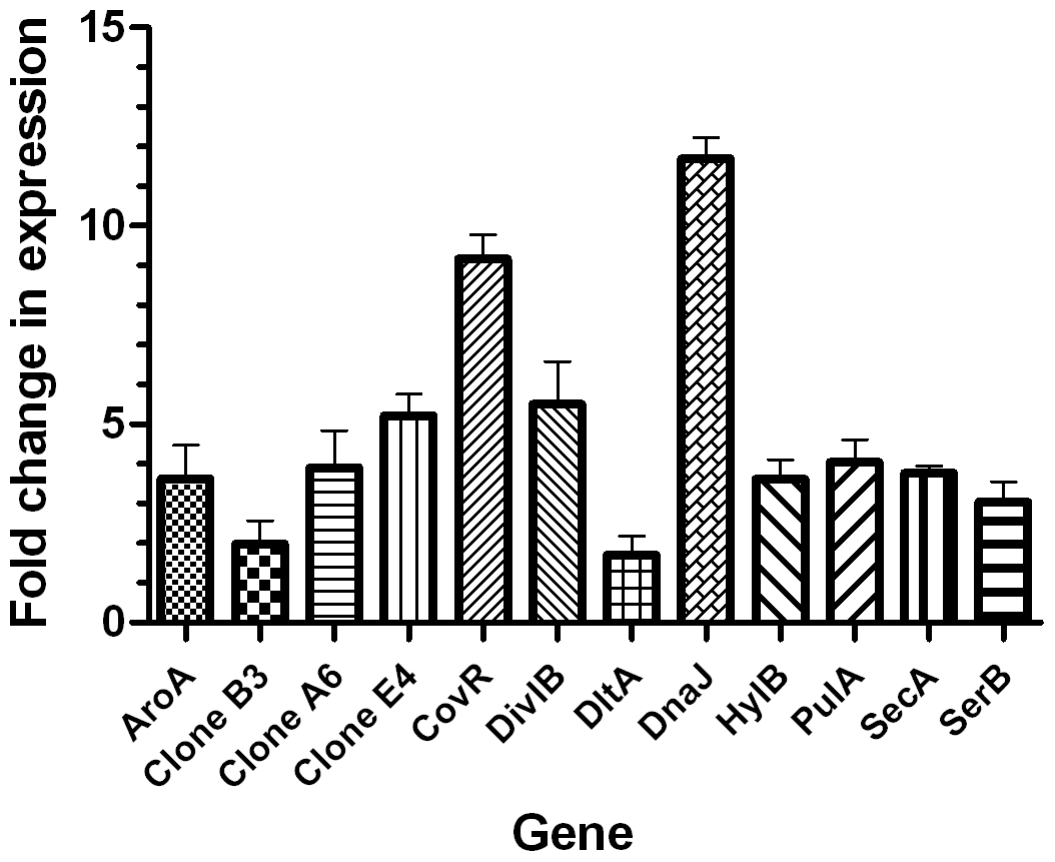
qRT-PCR determination of the genes up-regulated during intracellular survival of *S. agalactiae*. Real-time RT-PCR analysis of differentially expressed genes of *S. agalactiae* vis a vis survival in RAW264.7 and in THB cultures. Twelve genes, *aroA*, clone B3 (peptidase), clone A6 (polysaccharide biosynthesis protein), clone E4 (oligopeptide ABC transporter oligopeptide -binding protein), *covR*, *divIB*, *dltA*, *dnaJ*, *hylB*, *pulA*, *secA* and *serB* were selected and amplified using real-time RT-PCR. Real time RT-PCR data for each gene is relative to that obtained for the 16S rRNA control. Data points represent the means ± SD of three independent experiments.

The SCOTS approach, as used in this study, should result in the identification of genes that are upregulated by *S. agalactiae* upon interaction with macrophages [24]. Therefore, to validate our SCOTS results, we used quantitative PCR (q-PCR) to measure the expression levels of the genes from Table 2. In order to ensure that one gene from each functional category (the genes of unknown functions were not included) was selected for Real-time PCR analysis, we randomly selected one from the functional category (≤5 genes), three (>5 and ≤10 genes), and five (>10 genes). Therefore, a total of 12 genes were tested. The q-PCR analysis for these selected genes showed that they were indeed upregulated by *S. agalactiae* upon interaction with RAW264.7 (Fig. 9), with changes ranging from 1.7 to 11.69 fold.

## Discussion

*S. agalactiae* is a recognized pathogen in global aquaculture and disease outbreaks have resulted in significant economic losses in fish. In the present study, an experimental infection of *S. agalactiae* GD201008-001, a strain isolated from moribund cultural tilapia in China, was performed in mice. We found that this bacterial strain was highly virulent (LD50 <10^1^ CFU). The infected mice developed clinical signs of central nervous system (CNS) infection characteristic of brain damage. And the pathological findings in the brains correlated with the clinical behavioural abnormalities, such as lethargy and loss of orientation. These findings supported that *S. agalactiae* was neurotropic as described earlier by Eldar *et al*. [7] and Abuseliana *et al*. [25].

Our understanding of the pathogenesis of GBS infection remains limited, particularly concerning the mechanisms by which this bacterial species travels and disseminates in the bloodstream to reach the central nervous system. Efforts should be made to characterize the underlying pathogenic mechanisms that are essential for crossing the BBB. Histological investigations in the giant Queensland grouper, *Epinephelus lanceolatus* (Bloch), and other wild fish have shown that macrophages may act as a vehicle for *S. agalactiae*, allowing the bacterium to cross the BBB and enter the CNS and thereby be more easily disseminated to other organs and tissues, manifesting as a bacterial septicemia [26]. For this reason, we used RAW264.7 murine macrophages as a model to study the interactions between *S. agalactiae* and macrophages. Our study showed that *S. agalactiae* could be phagocytosed in large numbers by murine macrophages in the absence of complement and antibodies in a dose dependent manner. GBS not only entered macrophages very efficiently but also survived intracellularly for more than 24 h, a period that *in vivo* would be more than sufficient to maintain a bacteremia required for developing meningitis [13]. Our results indicated that the numbers of viable intracellular GBS in RAW264.7 decreased at first, then increased slightly up to 7 h.p.i., and then descended again afterwards. These dynamic changes probably reveal the process of interaction between GBS and macrophages. However, in general, the survival of the bacteria showed a downward trend. Mancuso *et al*. [27] showed that encapsulated GBS survived longer in mouse bone marrow-derived dendritic cells compared to mouse macrophages. The present study also showed that macrophages were injured by *S. agalactiae*. It is possible *S. agalactiae* may induce apoptosis or necrosis in macrophages, enabling the bacterium to overcome and then inhibit the host's immune defense system. Studies on murine macrophages have shown that human *S. agalactiae* induces macrophage apoptosis, affording a novel way to overcome the host immune system [28]. Liu *et al*. [12] have reported that β-hemolysin/cytolysin of GBS could produce direct cytolytic injury to macrophages and induce macrophage apoptosis over a longer interval. This led to us to speculate that hemolysin may play an important role in the mechanism of killing phagocytes in *S. agalactiae* GD201008-001. Nevertheless, this hypothesis remains to be defined in future studies.

Using the SCOTS differential cDNA cloning approach, we identified 60 differentially expressed *S. agalactiae* transcripts and q-PCR analysis showed that the genes were indeed upregulated upon interaction with RAW264.7 murine macrophages. Some of the genes identified by SCOTS might be considered, on the basis of their functions in other organisms, potential candidates as *S. agalactiae* virulence factors. For instance, the *dltA* gene (*dltA* belongs to an operon comprising four genes, *dltA*, *dltB*, *dltC*, and *dltD*), which is present in all genomes of low G+C bacteria determined so far [29]. In all species where this operon has been studied, all four of the genes are required to catalyze the incorporation of D-alanine residues into lipoteichoic acids (LTAs). D-alanylation of LTAs allows gram-positive bacteria to modulate their surface charge, to regulate ligand binding, and to control the electromechanical properties of the cell wall [29]. In addition, formation of D-alanyl-LTAs is required to resist the action of antimicrobial peptides in *L. monocytogenes* [30], *S. pneumoniae* [31], *Staphylococcus aureus* [32] and *S. pyogenes* (group A Streptococcus) [33]. The *S. agalactiae* D-alanyl-LTAs has been shown to have a role in resistance to defensins and phagocytic cells [34]. Therefore, *S. agalactiae* might modulate the degree of D-alanylation of its LTAs by upregulating *dltA* upon interaction with murine macrophages.

Limia A *et al*. [35] have shown that survival within phagocytic cells is associated with significant abnormalities in the maturation of phagosomes and trafficking in the host cell. Their data suggest the intracellular life of *M. avium* results in translocation of proteins by the Sec pathway [35]. Studies by Owens et al. [36] have shown that *secA* is critical to the protein export process in other bacteria such as *M. tuberculosis*. In this regard, it may be of interest to further evaluate the hypothesis that *secA* plays an important role in survival in macrophages by *S. agalactiae* by interfering with phagosome maturation.

It is known that all pathogens engulfed by macrophages suffer a barrage of bactericidal attacks from exposure to reactive oxygen and nitrogen species, antimicrobial peptides, proteases, peptidoglycan-degrading enzymes, and metal deprivation. In this study, *ciaH*, encoding the histidine kinase involved in many stress responses, was upregulated. Gene expression in prokaryotes is controlled by two-component systems that comprise a sensor histidine kinase and a cognate DNA binding response regulator. Pathogenic bacteria utilize two-component systems to regulate expression of their virulence factors and for adaptive responses to the external environment [37]. Qi *et al*. [38] reported that inactivation of *ciaH* in *S. mutans* abolished mutacin production, diminished competence development, altered sucrose-dependent biofilm formation, and significantly reduced acid tolerance. *CiaH* and *ciaR* are in the same genomic organization as the *ciaRH* operon in *S. pneumoniae* [39]. It has been reported that the two-component regulatory system CiaRH of *S. pneumoniae* affects β-lactam susceptibility, autolysis, bacteriocin production, competence development, host colonization and virulence [39]. It might be interesting to evaluate whether this sensor contributes to the adaption to the external environment.

Additionally, in this study we identified several upregulated genes of enzymes involved in the stress response, such as *clpE*, *nox*, *carB* and *msrB*. ATP-dependent caseinolytic proteases (Clp) play a fundamental role in stress tolerance and virulence in many pathogenic bacteria. The results of Zhang *et al*. [40] indicated that Clp ATPase (*clpE*) affected pneumococcal pathogenesis by modulating the expression of some important virulence determinants and metabolism-related factors in *S. pneumoniae*. Nair *et al*. [41] indicated that *clpE* is a novel member of the HSP100 family, involved in cell division and virulence in *L. monocytogenes*. The *nox* gene, encoding NADH oxidase, was previously reported to play an important role in the oxidative and acid stress responses of *S. mutans* [42]. *CarB* encodes carbamoyl-phosphate synthase. Park *et al*. [43] used a hypersensitive *Escherichia coli* genetic system to identify genes involved in resistance to nitrosative stress imposed by reactive nitrogen intermediates; one of the ten candidate genes was a carbamoyl-phosphate synthase. Methionine sulfoxide reductases A and B (*msrA* and *msrB*) are antioxidant repair enzymes that reduce *S*- and *R*-diastereomers of methionine sulfoxides back to methionine, respectively. Zhao *et al*. [44] reported that Msr repair enzymes are important for the oxidative stress response, macrophage survival, and persistent infection with *Enterococcus faecalis*. Ehrt *et al*. [45] suggested that *msrA* and *msrB* contribute to the resistance of *M. tuberculosis* against reactive nitrogen intermediates (RNI) and reactive oxygen intermediates (ROI). Msr can repair oxidative damage by reducing methionine sulfoxide (MetSO) back to methionine (Met) using electrons derived from thioredoxin, thioredoxin reductase, and NADPH [44]. We also found there is a thioredoxin reductase region in the clone C2 protein (pyridine nucleotide-disulfide oxidoreductase family protein) by using the BLAST search from NCBI (data not shown). Increased *clpE*, *nox*, *CarB* and *msrB* mRNA levels suggest that the encoded enzymes may be involved in the maintenance of function in stress response to adverse environmental conditions.

Interestingly, one of the genes identified by SCOTS (clone B1), *hylB*, encodes hyaluronate (HA) lyase. The degradation of polymeric HA and other glycosaminoglycans by HA lyases plays an essential role in many biological processes. For example, HA lyases can help *S. agalactiae* spread through host tissues [9]. It is thus tempting to speculate that upregulating the expression of the HA lyases identified in this work might, *in vivo*, be useful to increase the permeability of the BBB and thereby contribute to the migration of *S. agalactiae* to the CNS. Another gene, *pulA,* encodes pullulanase. It has previously been reported that *pulA* is likely to be involved in bacterial adhesion to host cells and suggested that *in vivo* immunization with this antigen could prevent streptococcal colonization [46], [47], [48], [49]. Gourlay *et al*. [50] also found that both recombinant forms of pullulanase from GBS appear to bind to ME180 cervical epithelial cells. Similarly, the *gap* gene encodes glyceraldehyde-3-phosphate dehydrogenase (GAPDH), possibly implicated in the adhesion of the bacteria. Brassard *et al*. [51] found the GAPDH protein of *S. suis* seemed to be involved in the first steps of bacterial adhesion to host cells. Also, Oliveira *et al*. provided evidence for a novel function of secreted GAPDH as an inducer of apoptosis in murine macrophages [52]. It might therefore be plausible that after GBS enters the CNS via a “Trojan horse” or “modified” Trojan horse mechanism, this bacterium may escape from the “Trojan horse” with the help of GAPDH and go on to promote the development of meningitis.

*CcpA* encodes catabolite control protein A. Willenborg *et al*. [53] demonstrated that *ccpA* has a significant effect on capsule synthesis and the virulence properties of *S. suis*. Deletion of *ccpA* altered expression of surface-associated virulence factors and revealed a markedly reduced thickness of the capsule. This phenotype correlated with enhanced binding to porcine plasma proteins and a reduced resistance to killing by porcine neutrophils. It is well accepted that the capsule of GBS enables avoidance of immunologic clearance by host phagocytic cells, including neutrophils and macrophages [9]. The role of *ccpA* during macrophage infection in GBS is unclear; however, considering that *ccpA* is involved in the formation of the capsule, we hypothesized that upregulation of *ccpA* expression may contribute to the resistance of *S. agalactiae* to phagocytosis and survival in RAW264.7 cells. Further studies are required to evaluate this hypothesis.

Regulatory systems play important roles in the adaptive responses of many bacteria to environmental cues such as cell contact and entry. *MerR*-type and *lysR*-type transcriptional regulators (LTTRs) regulate a diverse set of genes, including those involved in virulence, metabolism, quorum sensing and motility [54], [55]. CovR is a response regulator protein. Recently, CovR/S two-component global regulatory system has received considerable attention. Sendi *et al*. [56] have shown the critical involvement of CovR/S in virulence of *S. agalactiae*. Mutations in the *cov* gene lead to an increased β-hemolytic activity with lower capsule expression. And the phenotype showed low intracellular survival in human neutrophils in contrast to the low hemolytic variant with high capsule expression. The findings of Cumley *et al*. [10] showed that CovS/R is required for survival of *S. agalactiae* inside the phagosome. A recent study by Sagar *et al.* [57] demonstrated that the absence of β-hemolysin may enable *S. agalactiae* to survive in higher numbers inside professional phagocytes, and highlighted the importance of CovR/S regulator due to its suppression effect on β-hemolysin expression. In addition, a recent publication reported that the expression of the *cov* regulator was increased in *S. pyogenes* recovered from the intracellular environment of macrophages [58]. *MerR*, *lysR* and *covR* were also found in our SCOTS result. Therefore, it is hypothesized that transcriptional regulators might play an important role in survival within phagosomes and hence the virulence of GBS.

Globally, our SCOTS results indicate that internalization of GBS by macrophages causes major changes in the expression of genes associated with signal transduction mechanisms and transcription, showing that GBS senses the phagosomal environment and activates mechanisms necessary for survival in a new niche. Metabolic adaptation accounts for more than 50% (32/60) of differentially regulated genes in intracellular bacteria. This agrees with the observation for other pathogens [59], [60] that some of the most highly expressed genes in intracellular bacteria are those involved in metabolism. The changes may reflect the ability of *S. agalactiae* to utilize resources present in macrophages.

In conclusion, we investigated the virulence of *S. agalactiae* isolated from fish in mouse and macrophage models. For the first time, we identified genes that were up-regulated during the interaction between GBS and macrophages. Characterizing these genes and their products and deciphering their roles in intracellular growth will enable us to gain a better understanding of *S. agalactiae* pathogenesis. Allele inactivation studies addressing the roles of several of these genes are now under way.

## Materials and Methods

### Ethics statement

Animal experiments were carried out according to animal welfare standards and approved by the Ethical Committee for Animal Experiments of Nanjing Agricultural University, China. All animal experiments complied with the guidelines of the Animal Welfare Council of China.

### Cell lines, bacterial strains, plasmids and culture conditions

The cell lines, bacterial strains and plasmids used in this study are listed in Table 3. A green fluorescent protein (GFP) expression plasmid, pSL5.28 [61], was provided by the Vaccine and Infectious Disease Organization, University of Saskatchewan. RAW264.7 cells (ATCC) were maintained in Dulbecco's modified Eagle medium (DMEM) with high glucose (Gibco, Invitrogen Corp., Carlsbad, CA) supplemented with 10% fetal bovine serum (FBS). Cells were used from passages 5 to 20.

**Table 3:** Characteristics of cell lines, bacterial strains, plasmids and primers used in this study.

Cell line, strain, plasmid or primer primer	Characteristics and/or sequences	Source/reference
**Cell line**		
RAW264.7	murine (BALB/c) macrophage cell line	ATCC
**Strains**		
GD20101008-001	*S.agalactiae*, serotype Ia, isolated from cultured tilapia in China.	Our lab
DH5α	*Escherichia coli*	TaKaRa
**Plasmids**		
pSL5.28	green fluorescent protein (GFP) expression	[61]
pMD18-T	Vector for cloning Taq polymerase-amplified PCR products	TaKaRa
pMD18-T16S	pMD18-T containing the 16S rRNA sequence (1507bp) of *S.agalactiae*	This work
pMD18-T23S1	pMD18-T containing the 5′end 1500 bp fragment of 23S rRNA of *S.agalactiae*	This work
pMD18-T23S2	pMD18-T containing the 3′end 1403 bp fragment of 23S rRNA of *S.agalactiae*	This work
**Primers**		
SCOTS-N6-01	5′ GACACTCTCGAGACATCACCGGTACCNNNNNN 3′	This work
SCOTS-N6-02	5′ TGCTCTAGACGTCCTGATGGTTCNNNNNN 3′	This work
SCOTS01	5′ GACACTCTCGAGACATCACCGGTACC 3′	This work
SCOTS02	5′ TGCTCTAGACGTCCTGATGGTTC 3′	This work
16S01	5′ AGAGTTTGATCCTGGCTCAGG 3′	
16S02	5′ TACCTTGTTACGACTTCACC 3′; amplifies the 16S rRNA sequence(1507 bp)	This work
23SN01	5′ GGTTAAGTTAATAAGGGCGCAC 3′	
23SN02	5′ TTGACCAGACACTTCCAATCGT 3′; amplifies the 5′ end of 23S rRNA sequence(1500 bp)	This work
23SC01	5′ ACAGTGAGGTGTGATATGAGTC 3′	
23SC02	5′ TTGGATAAGTCCTCGAGCTATT 3′; amplifies the 3′ end of 23S rRNA sequence(1403 bp)	This work
16S-L	5′CGACGATACATAGCCGACCT3′	
16S-R	5′CCGTCACTTGGTAGATTTTCC3′; amplifies *S.agalactiae* 16S rRNA sequence (210 bp)	This work
AroA-L	5′GATCGGATTCAGGTCGTTGT3′	
AroA-R	5′CTTTGACCAAGAGAGCAGCA3′; amplifies the sequence of *aroA* (172 bp)	This work
Clone B3-L	5′CAATCAAGCTCTCAGCCAAA3′	
Clone B3-R	5′GTGGTATGAATGGCGTCGTA3′; amplifies the sequence of clone B3 (210 bp)	This work
Clone A6-L	5′ATTGCGGGAGTGGTAGGAGTT3′	
Clone A6-R	5′TGTACGTCTTGAAGCCAGTAG3′; amplifies the sequence of clone A6 (162 bp)	This work
Clone E4-L	5′CGTCAGCAGTAACAGGCTCA3′	
Clone E4-R	5′CGATCCAAAGGACCGTTATG3′; amplifies the sequence of clone E4 (194 bp)	This work
CvoR-L	5′TCCAATAAATGTTCGCGTGTC3′	
CvoR-R	5′CAGCTGTAGCAGAAGAAAGTG3′; amplifies the sequence of clone *covR* (189 bp)	This work
DivIB-L	5′GACTATTTTCCGCAACCTGAC3′	
DivIB-R	5′AGAAAGACTTCCTTTTTACAA3′; amplifies the sequence of clone *divIB* (185 bp)	This work
DltA-L	5′GTTCCTAACATTTCATCAGTA3′	
DltA-R	5′GCTACTATCTAACGATTTCAA3′; amplifies the sequence of clone *dltA*(198 bp)	This work
DnaJ-L	5′TCAATGGTGGGCTTTATGGT3′	
DnaJ-R	5′TGACGTTTCTACCGCTCCAT3′; amplifies the sequence of clone *dnaJ*(169 bp)	This work
HylB-L	5′CGCTACTTATCGTCGTTTGGA3′	
HylB-R	5′ATTGAGCGAGGGACACCGATT3′; amplifies the sequence of clone *hylB* (195 bp)	This work
PulA-L	5′CGAGAAGCAACATATTTACCG3′	
PulA-R	5′TAACTTGGTCTGGGGCATTGT3′; amplifies the sequence of clone *pulA* (188 bp)	This work
SecA-L	5′AGATAACTCCCAGTGCTAAAC3′	
SecA-R	5′AACTGAAGATACTTATCGCCC3′; amplifies the sequence of clone *secA*(206 bp)	This work
SerB-L	5′TCCTACCCCAGCGGATTTGAT3′	
SerB-R	5′AAAGCGAATCGTCCTGGTGTC3′; amplifies the sequence of clone *serB*(189 bp)	This work

*S. agalactiae* strain GD201008-001, β-hemolysin/cytolysin positive, which belongs to serotype Ia, MLST type ST-7, was isolated from farmed tilapia with meningoencephalitis in the Guangdong province of China in 2010 [62]. Its genome sequence has been deposited in the GenBank database under accession number CP003810. The bacterial strain was grown using either Todd-Hewitt broth (THB) or agar (THA) (Becton Dickinson, MD, USA) or sheep blood agar plates at 37°C.

### Determination of virulence of GD201008-001

BALB/c mice (22–24 g, 50 days old) were purchased from the Experimental Animal Center, Yangzhou University. The mice were divided into 5 groups with 10 mice in each group. Mid-exponential phase bacteria were washed twice in PBS. Bacteria were then resuspended in PBS to 1×10^5^ CFU/ml and a 10× dilution serial with the lowest concentration being 1×10^2^ CFU/ml was prepared. Four groups of mice were injected intraperitoneally (i.p) with 100 µl of the bacterial suspension of different dilutions respectively. The 5th group was injected with 100 µl PBS and served as control. Death of mice was recorded.

### Experimental infection of mice

BALB/c mice (22–24 g, 50 days old) were divided into 6 groups with 5 mice in each group. Mid-exponential phase bacteria were washed twice in PBS. Bacteria were then resuspended in PBS to 2×10^3^ CFU/ml. Five groups of mice were injected i.p with 100 µl of the bacterial suspension. The 6th group was injected with 100 µl PBS and served as control. Samples were taken at 3 h, 6 h, 12 h and 20 h post infection. At each designated time, one mouse from each infection group and one from the control group were anesthetized with CO_2_. Blood was collected and the brains, livers, and spleens were obtained aseptically. In order to avoid possible surface contamination, livers, and spleens were sterilized with 75% alcohol,and then washed with PBS. The organs (0.05 g/organ) were trimmed, placed in 500 µl of PBS, and homogenized in a high-speed homogenizer. Then, 200 µl of 10^2^, 10^3^, 10^4^ or 10^5^ dilutions of the homogenate in PBS were plated onto blood agar plates. Blood samples (200 µl) were also plated. The blood agar plates were incubated overnight at 37°C. Colonies were counted and expressed as CFU/g for organ samples or CFU/ml for blood samples.

### Histopathology and immunohistochemistry

At 20 h.p.i., the infected mice were euthanized and the brains was collected and placed into 10% neutral buffered formalin. After fixation the organs were embedded in paraffin, 4 mm sections were taken, and these were stained with hematoxylin and eosin for histological evaluation. For immunohistochemistry, sequential slides were stained using an immunoperoxidase method as follows. First, sections were blocked with 3% H_2_O_2_ for 30 min, and then non-specific background staining was blocked by incubating the sections for 30 min with normal rabbit serum. The sections were then incubated for 1 h with rabbit anti-GBS polyclonal serum (1∶1000, prepared in our laboratory using the GBS strain GD201008-001 from this study) or unimmunized rabbit serum (1∶1000) as a control, followed by biotinylated goat anti-rabbit immunoglobulin (Ding-Guo, China) diluted 1/100 at 37°C for 30 min and subsequent incubation with HRP conjugated streptavidin (DingGuo, China) at 37°C for 30 min. Finally, the sections were developed with the HRP-DAB chromogenic substrate kit (Tiangen, China) for 10 min, and then the slides were counterstained with hematoxylin.

### Phagocytosis and intracellular survival assays

Green fluorescent protein (GFP)-expressing *S. agalactiae* GD201008-001 was prepared as previously described [61]. The phagocytosis assay was performed as described above, with some modifications. RAW264.7 cells were grown on 24-well tissue culture plates. After an initial 1 h exposure to the bacteria (at an MOI of 1, 10 or 100), gentamicin and penicillin were added. After antibiotic treatment, the cell monolayers were washed three times and the medium was replaced with 1 ml of sterile distilled water to lyse the macrophages. After vigorous pipetting to ensure complete cell lysis, viable intracellular streptococci were determined by quantitative plating of serial dilutions of the lysates on THB agar. After overnight incubation at 37°C, the number of bacteria was calculated.

For intracellular survival studies, an internalization assay was performed as described above, except that after a 1 h initial exposure to the bacteria, gentamicin and penicillin were added and the treatment was continued for different times up to 24 h. At different time points, cells were processed as described above and bacteria counted. The results come from at least three independent experiments.

### Cellular injury assays

A lactate dehydrogenase (LDH) release measurement assay was used to measure cytotoxicity levels (Promega CytoTox96, Promega Corporation, Madison, WI, USA) as previously described [63] with some modifications. Briefly, bacteria were grown and diluted as described above, and cells grown in 96-well plates were infected with 100 µL aliquots of a bacterial suspension at different concentrations (MOIs of 1, 10 or 100). The plates were centrifuged at 800×g for 10 min to bring the bacteria to the surface of each monolayer and were then incubated for 4 h at 37°C with 5% CO_2_ (see Results). Non-infected cells and bacteria alone were used as negative controls, whereas cells lysed with the lysis solution from the Promega CytoTox96 kit (Promega Corporation, Madison, WI) were used as positive controls (100% toxicity). At the end of the incubation time, the plates were centrifuged at 300 g for 20 min to pellet the bacteria and the LDH concentration was measured as previously described [64].

### Experimental infection, RNA isolation, cDNA synthesis and amplification

For RNA isolation, macrophage infection was performed as described above with some modifications. Cells were seeded at 2×10^6^ cells per well in 6-well tissue culture dishes. Bacteria were grown in THB to log-phase, washed in PBS and then added to the cell monolayer at an MOI of 100∶1 before centrifugation for 10 min at 800×g to synchronize phagocytosis. After incubation and antibiotic treatment, total RNA was isolated from the infected cells or from broth-grown bacteria using TRIzol reagent (Invitrogen) according to the manufacturer's instructions. RNA samples were treated with RNase-free DNase I (Fermentas) and RNA concentrations and integrity were evaluated by A260/A280 spectrophotometer readings and agarose gel electrophoresis, respectively. Total RNA isolated from infected macrophages or from broth-grown bacteria was converted to first strand cDNA by random priming with Superscript II reverse transcriptase (Invitrogen). Random priming was performed as described [66] using oligonucleotides containing the SCOTS-N6-01 and SCOTS-N6-02 terminal sequences at the 5′ end for cDNAs from the infected macrophages and from broth-grown bacteria, respectively, and a random hexamer at the 3′ end. The cDNA was then converted to double-stranded form with Klenow fragment (Fermentas) as described previously [65]. Then, individual cDNAs were amplified by PCR with 30 cycles of amplification (95°C for 30 s, 66°C for 60 s and 72°C for 60 s).

### Selective capture of transcribed sequences (SCOTS)

Genomic DNA from GD20101008-001 was biotinylated as described previously [22]. The biotinylated *S. agalactiae* genomic DNA (12 µg) was mixed with 100 µg of pMD18-T16S, pMD18-T23S1 and pMD18-T23S2 plasmid DNA (*S. agalactiae rrnA* DNA cloned into pMD18-T). The mixture was sonicated using a microprobe for 5 s at an output power of 25 W (Fisher Scientific 100). After sonication, the mixture was precipitated with 1/10 volume of 3 M sodium acetate and an equal volume of isopropanol, and re-suspended in 160 µl of 10 mM N-(2-hydroxyethyl) piperazine-N-(3- propanesulfonic acid) (EPPS)-1 mM EDTA. The mixture was then divided into 20 samples of 8 µl each. For each round of SCOTS, a sample of the mixture (containing 0.6 µg of *S. agalactiae* genomic DNA and 5 µg of *rrnA* DNA) was denatured by incubation at 98°C for 3 min. Two microlitres of 1 M NaCl were added to the mixture and it was incubated at 67°C for 30 min. This step allowed the plasmid *rrnA* DNA to hybridize to the *rrnA* sites on the *S. agalactiae* genomic DNA, thereby rendering these sites unavailable for hybridization with ribosomal DNA present in the cDNA mixtures. At the same time, total amplified cDNA, either from infected cells or from broth grown bacteria in 8 µl of 10 mM EPPS-1 mM EDTA was also denatured at 98°C for 3 min followed by the addition of 2 µl of 1 M NaCl. The denatured cDNA mixture was added to the biotinylated chromosomal DNA–*rrnA* pre-hybridized mixture, and hybridization was continued at 67°C for 24 h. cDNA was captured by streptavidin-coated magnetic beads (Dynal M280) according to the manufacturer's instructions. The process of elution was performed as described previously [22]. For each growth condition, in the first round of SCOTS, 10 separate samples of the cDNA mixtures were captured by hybridization to rDNA-blocked genomic DNA in parallel reactions.

### Enrichment for cDNA molecules in macrophages

To identify cDNA molecules representing transcripts from genes whose expression is either specific to or upregulated during the interaction between *S. agalactiae* and macrophages, an additional step was included in the experiments. Preparations of cDNA mixtures from *S. agalactiae* that had interacted with macrophages were obtained by three rounds of SCOTS and added to biotinylated genomic DNA that had been pre-hybridized with both rDNA and cDNA preparations from broth-grown *S. agalactiae* (also obtained following three rounds of SCOTS). Hybridization proceeded for 24 h at 67°C, and the hybridized molecules were recovered by binding to streptavidin-coated beads as described above.

### Dot blot hybridization and analysis of individual cDNA clones

Macrophage-specific or upregulated cDNAs were ligated into the cloning vector pMD18-T (TaKaRa). For verification, cloned inserts were amplified by PCR. PCR amplicons of positive SCOTS clones were transferred to a positively charged membrane (Roche). Samples of *S. agalactiae* genomic DNA and cDNA mixtures generated from infected macrophages and bacteria cultured under normal growth conditions were labeled with DIG-dUTP (Roche) and used as probes. Dot blot hybridization using DIG Easy Hyb (Roche) was used in accordance with the manufacturer's instructions. *S. agalactiae* genomic DNA (1 or 0.1 µg) spotted on nylon membranes was used as a positive control exposed alongside the corresponding SCOTS clone dot blots. Hybridization signals stronger than 1 µg of genomic DNA or weaker than 0.1 µg of genomic DNA were deemed strong or weak signals, respectively. The positive sequences of these clones satisfied the requirement that their signals be weaker or absent when hybridized to normalized culture-specific cDNA, and strong on the blot hybridizing to normalized macrophage-specific cDNA, indicating that these sequences are either absent or present in much less abundance in cDNA prepared from *S. agalactiae* grown under standard culture conditions. The inserts of positive cDNA clones from the library were sequenced using the standard Sanger method. Database searches as well as DNA and protein similarity comparisons were carried out using the BLAST algorithm from the National Center for Biotechnology Information at the National Library of Medicine. http://www.ncbi.nlm.nih.gov/BLAST/Blast.cgi).

### Real-time qRT-PCR

To validate the SCOTS results, we used quantitative PCR (q-PCR) to measure the level of expression of randomly selected genes on a new series of infected replicates. Infection of RAW264.7, growth of *S. agalactiae* in broth, and RNA extraction from both samples were performed as described above. cDNAs were synthesized in triplicate using Super-Script II with random hexamers (Roche, Laval, Quebec,Canada). q-PCR was performed using the QuantiTect SybrGreen PCR kit (QIAGEN, Mississauga, Ontario, Canada) according to the manufacturer's instructions. For each sample, a no-reverse transcription reaction was run as a control. The primers used are described in Table 1. For each q-PCR run, the calculated threshold cycle (CT) was normalized to the CT of the 16S rRNA internal control amplified from the corresponding sample, and the n-fold change was calculated by the 2^−ΔΔCT^ method as previously described [66].

### Statistical analysis

Each cell internalization or injury experiment was repeated in at least three independent test series with a minimum of four parallels each. All statistics were performed using an unpaired two-tailed t-test with a 95% confidence interval. A *P*-value <0.05 was considered to indicate a significant difference. In all experiments, error bars were denoted using the standard deviation (±SD) of independent experiments.
